# Myeloma therapy: pursuing the plasma cell

**DOI:** 10.1038/sj.bjc.6605710

**Published:** 2010-06-29

**Authors:** J Cavenagh

**Affiliations:** 1 Department Haematology, St Bartholomew's Hospital, London, EC1A 7BE, UK

Sagar Lonial is to be heartily congratulated on having brought together an impressive array of established leaders in the field of multiple myeloma (MM) to produce a comprehensive review of this difficult disease and its management.

The book is divided into seven sections dealing with all the major areas relevant to MM. The first section addresses ‘Biological considerations and multiple myeloma’. The chapter relating to the ‘Epidemiology of MM’ (A Langston and D Francis) is a thoughtful summary of current understanding of this topic and discusses the intriguing issue of familial predisposition to MM, which is, as yet, unexplained, but clearly has significant importance to the pathophysiology of MM. The chapter that focuses on the tumour microenvironment (M Raab and K Anderson) nicely summarises the current state of knowledge concerning the inter-dependency of MM tumour cells and the bone marrow microenvironment. This is a current paradigm, championed by Professor Anderson's group at the Dana-Farber that has underpinned the development of the so-called ‘novel therapies’, which has led to the significant advances in MM treatment. The chapter on cytogenetics (E Braggio, M Sebag and R Fonseca) is a detailed and thorough overview of the cytogenetic abnormalities found in MM and of their prognostic significance.

The second section of the book addresses ‘conventional’ cytotoxic treatments. The chapters by J-L Harousseau and A Palumbo are typically authoritative and logical overviews of the function of autologous stem cell transplantation and therapies for non-transplant eligible patients, respectively. A chapter by S Ailawadhi and A Chanan-Khan discusses the function of anthracyclines in MM and reminds us that ‘conventional’ drugs still have activity in this disease and should not be discarded from the anti-MM armamentarium.

The third section addresses the immune-based therapies in MM as well as comprehensive reviews about immunotherapy and antibody-based approaches, and the function of allogeneic transplantation is discussed (F Wang and E Waller). This issue remains, arguably, one of the most important in the treatment of MM. The chapter summarises the current data and we are left in no doubt that further work is required to define the role of this treatment in MM.

Section four, in over 60 pages, comprises a ‘state of the art’ overview of the data concerning the ‘novel agents’: thalidomide, lenalidomide and bortezomib. Each chapter is authored by recognised experts in the field and describes the early development and later definitive clinical trial data that has led to the establishment of these agents into frontline MM care. Population-based data have shown that coincident with the advent of the ‘novel agents’, there has been the single most important increment in outcome for patients with MM. Clearly, the next questions will involve issues concerning the most appropriate sequencing of these drugs, the most effective combinations, the continuing role for ASCT in the ‘novel agent era’ and the best selection of therapy for specific groups of patients.

Section five addresses ‘Current and Future Targets’. This is arguably the most stimulating section of the book and different chapters discuss, among others, hsp90, PI3K/Akt, mTOR, FGFR3 and HDAC inhibitors. Each chapter has been written by experts in the field and offer clear and insightful reviews of these emerging potential targets for therapy. For example, the chapter by C Mitsiades on hsp90 ranges from scientific rationale to early phase clinical trials in the area. The authors offer the hope that new-targeted therapies will be developed and describe the strong rationale for combining these agents with pre-existing ones (e.g. hsp90 inhibitors with bortezomib). Let us hope that these scientifically designed combinations will further improve outcomes for patients with MM.

Section six addresses the most important area of supportive care in MM. The issues discussed include bone disease and its treatment with kyphoplasy and vertebroplasty, anaemia, functional imaging and renal impairment. The final section addresses ‘Other Plasma Cell Disorders’. These chapters comprise authoritative reviews on Waldenstrom's Macroglobulinemia, AL Amyloidosis, the mysterious POEMS syndrome and MGUS. Penned by recognised experts in the field, these chapters represent an excellent resource and reference.

Overall, the book is an outstanding resource for clinicians working in the area of MM, which will have long-lasting value and relevance.

